# ﻿*Dyscrituluseuropaeus* sp. nov. (Hymenoptera, Braconidae, Aphidiinae): description of a new aphid parasitoid species with an identification key for species of the genus

**DOI:** 10.3897/zookeys.1175.106416

**Published:** 2023-08-18

**Authors:** Korana Kocić, Andjeljko Petrović, Jelisaveta Čkrkić, Cornelis van Achterberg, Željko Tomanović

**Affiliations:** 1 Institute of Zoology, Faculty of Biology, University of Belgrade, Studentski Trg 16, 11000 Belgrade, Serbia University of Belgrade Belgrade Serbia; 2 Centre for Biodiversity Genomics, University of Guelph, 50 Stone Road, N1G 2W1, Guelph, Ontario, Canada University of Guelph Guelph Canada; 3 Naturalis Biodiversity Center, P.O. 9517, 2300 RA Leiden, Netherlands Naturalis Biodiversity Center Leiden Netherlands; 4 Serbian Academy of Sciences and Arts, Knez Mihailova 35, 11000, Belgrade, Serbia Serbian Academy of Sciences and Arts Belgrade Serbia

**Keywords:** Aphid parasitoids, Europe, museum collections, new species, taxonomy

## Abstract

The braconid genus *Dyscritulus* Hincks is a small member of the subfamily Aphidiinae, distributed in Europe and Central Asia. All its species are highly specialized parasitoids of aphids of the genera *Drepanosiphum* Koch and, probably, *Periphyllus* van der Hoeven which are mostly associated with maple and sycamore trees (genus *Acer*). Upon examination of specimens from the Naturalis Biodiversity Center, Leiden, we unexpectedly noted unusual variability in morphological characters compared to other known *Dyscritulus* species. Further inspection of other material previously identified as *Dyscritulusplaniceps* Marshall, 1896 revealed additional specimens with the same morphological variability. Here we describe a new species of the genus, *Dyscrituluseuropaeus***sp. nov.**, associated with *Drepanosiphum* aphids on *Acer*.

## ﻿Introduction

Genus *Dyscritulus* Hincks, 1943 is classified within the tribe Praini, together with five other genera, *Praon* Haliday, 1833, *Areopraon* Mackauer, 1959, *Choreopraon* Mackauer, 2000, *Pseudopraon* Starý, 1975 and *Astigmapraon* Tian & Chen, 2017. With only four currently described species (*Dyscritulusplaniceps* Marshall, 1896, *D.pygmaeus* Mackauer, 1961, *D.trjapitzini* Davidian, 2018 and *D.dzhungaricus* Davidian, 2019), it is a small member of the subfamily Aphidiinae.

When *Dyscritulus* was first described, it bore a different generic name. Marshall (1896) described the genus *Dyscritus* Marshall, 1896, with the monotypic species *Dyscritusplaniceps* Marshall, 1896 (Viereck 1914). Morley (1933) described an additional species, *Dyscritussuffolciensis* Morley, 1933. However, almost half a century after the first mention of the genus, William Hincks (1943) changed the generic name to *Dyscritulus* Hincks, 1943; reason being that the name *Dyscritus* existed prior to the Marshall`s description (*Dyscritus* Scudder, 1868). Starý (1959) wrote a detailed revision of the genus and its species *D.planiceps*, noting that *D.suffolciensis* is incorrectly placed and that, according to Hincks, it is not a member of the Aphidiinae subfamily. Subsequently, three additional species were described: *Dyscrituluspygmaeus*, *D.trjapitzini* and *D.dzhungaricus*.

Distributed in Europe and Central Asia (Starý 1959; Davidian 2018, 2019, 2020), *Dyscritulus* species are parasitoids of the aphid genera *Drepanosiphum* Koch and *Periphyllus* van der Hoeven, which are almost exclusively associated with sycamore and maple species of *Acer* L. However, *Periphyllus* aphids are associated with specific parasitoid complexes [*Aphidiussetiger* Mackauer, *Areopraonsilvestre* (Starý) and *Trioxysfalcatus* Mackauer] (Starý 1972) and records of parasitization by *D.planiceps* (Davidian 2018) should be re-evaluated. Only the biology of *D.planiceps* has been studied and is all that is known at the moment. Starý (1970) stated that *D.planiceps* prefers to parasitize alatаe adults of *Drepanosiphumplatanoidis* Schrank over other aphid development stages. Being highly specialized, the seasonal activity of *Dyscritulus* parasitoids coincides with the seasonal activity of its aphid host, i.e., they enter a diapause period at the same time (Starý 1970). As in *Praon*, the pupation is always external, which is hypothesized to be a secondary adaptation to avoid hyperparasitism (Mackauer 1961; Tomanović et al. 2006). The cocoon is disc-shaped, spun beneath the empty aphids’ remains, more or less flat except for the outer edges and the middle portion, which is firmly attached to the dead aphids remains.

After examining *Dyscritulus* specimens from Naturalis Biodiversity Center, Leiden, we unexpectedly found an unusual variability in morphological characters compared to other known *Dyscritulus* species. Here we describe a new species with European distribution, *Dyscrituluseuropaeus* sp. nov., a parasitoid of *Drepanosiphum* aphids on *Acer* trees. Additionally, we provide a key to the identification of all currently known species of *Dyscritulus* and further discuss their taxonomy and the importance of museum collections for biodiversity research.

## ﻿Materials and methods

The specimens examined in this study were collected in Spain, France and Serbia. Two females from Spain (Málaga) and one from France (Mt Ventoux) are from the collection of Naturalis Biodiversity Center, labelled without a specified sampling method, but were collected by using a sweep net. The remaining specimens from Serbia were collected by rearing during 2006–2013 and are deposited in the collection of University of Belgrade, Serbia (Faculty of Biology, Institute of Zoology). Plant material with aphid colonies was kept in plastic containers covered by mesh for several weeks, under laboratory conditions, until the emergence of parasitoids. Live aphids were preserved in 96% ethanol for further identification. Parasitoids were either transferred to 96% ethanol or dry mounted. After examination under a ZEISS Discovery V8 stereomicroscope (Carl Zeiss MicroImaging GmbH, Göttingen, Germany), specimens were dissected and slide mounted in Berlese medium. Photographs of the dissected specimens were taken with a Leica DM LS phase contrast microscope (Leica Microsystems GmbH, Wetzlar, Germany). The obtained photographs were stacked in Helicon Focus software (v. 7.6.1; www.heliconsoft.com). ImageJ software (Schneider et al. 2012) was used to measure all important taxonomical characters. Morphological terminology follows Sharkey and Wharton (1997).

## ﻿Results

### ﻿Description of a new species

#### ﻿ Dyscritulus europaeus

Taxon classificationAnimaliaHymenopteraBraconidae

Kocić & Tomanović sp. nov.

7B4F33D2-2FF5-52DB-B298-9E75C6312319

https://zoobank.org/2F9F79F5-3F7B-45A1-8EA3-8FACA4D0FE98

**Figure 1.**
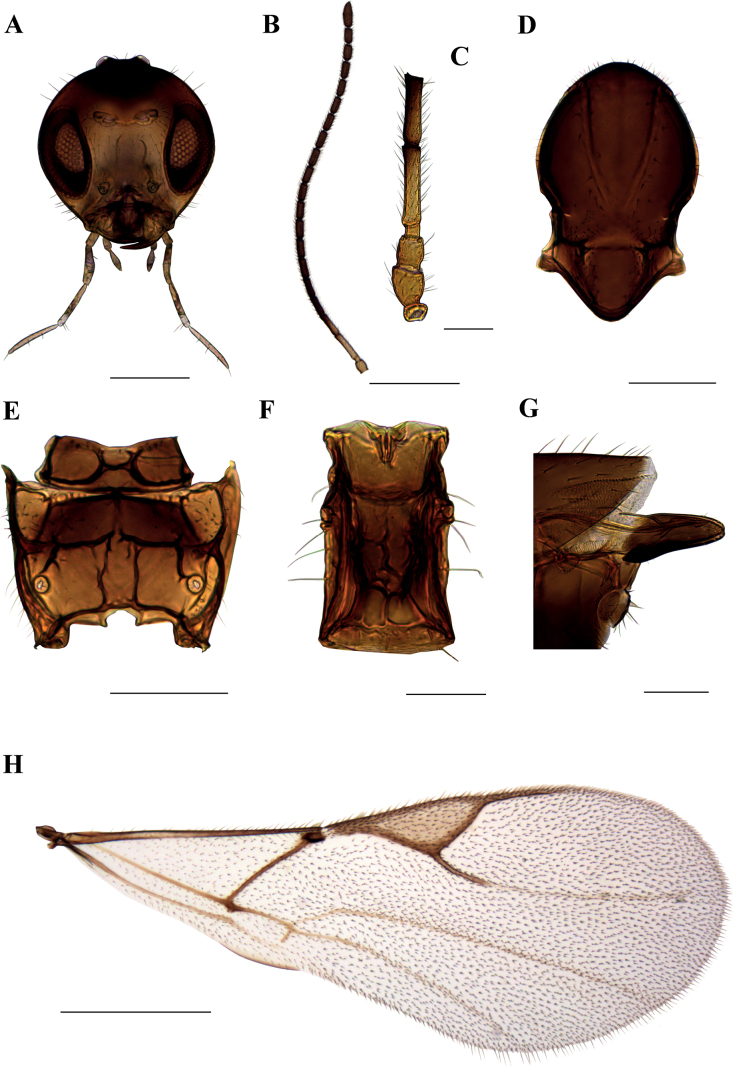
*Dyscrituluseuropaeus* sp. nov. holotype female **A** head **B** antenna **C** scape, pedicel, first and second flagellomere **D** mesonotum (=mesoscutum) – dorsal aspect **E** propodeum – dorsal aspect **F** petiole – dorsal aspect **G** ovipositor sheaths – lateral aspect **H** fore wing. Scale bars: 200 µm (**A**, **D**, **E**); 500 µm (**B**, **H**); 100 µm (**C**, **F**, **G**).

**Figure 2.**
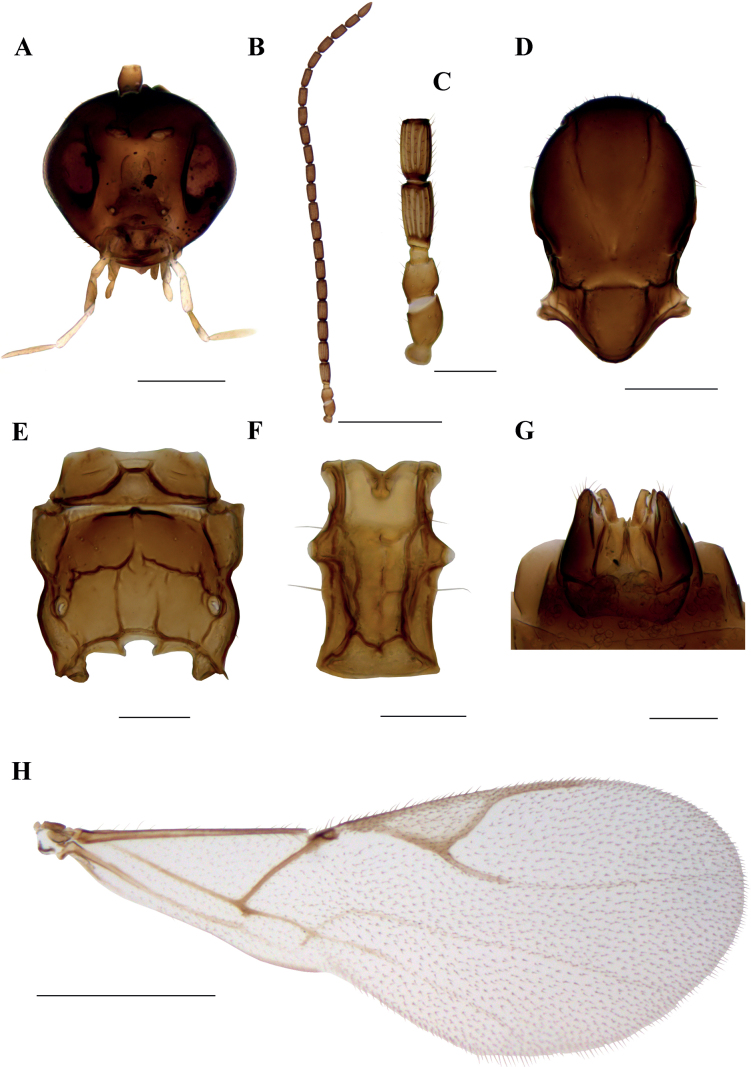
*Dyscrituluseuropaeus* sp. nov. paratype male **A** head **B** antenna **C** scape, pedicel, first and second flagellomere **D** mesonotum (=mesoscutum) – dorsal aspect **E** propodeum – dorsal aspect **F** petiole – dorsal aspect **G** genitalia – ventral aspect **H** fore wing. Scale bars: 200 µm (**A**, **D**); 500 µm (**B**, **H**); 100 µm (**C**, **E**, **F**, **G**).

**Figure 3.**
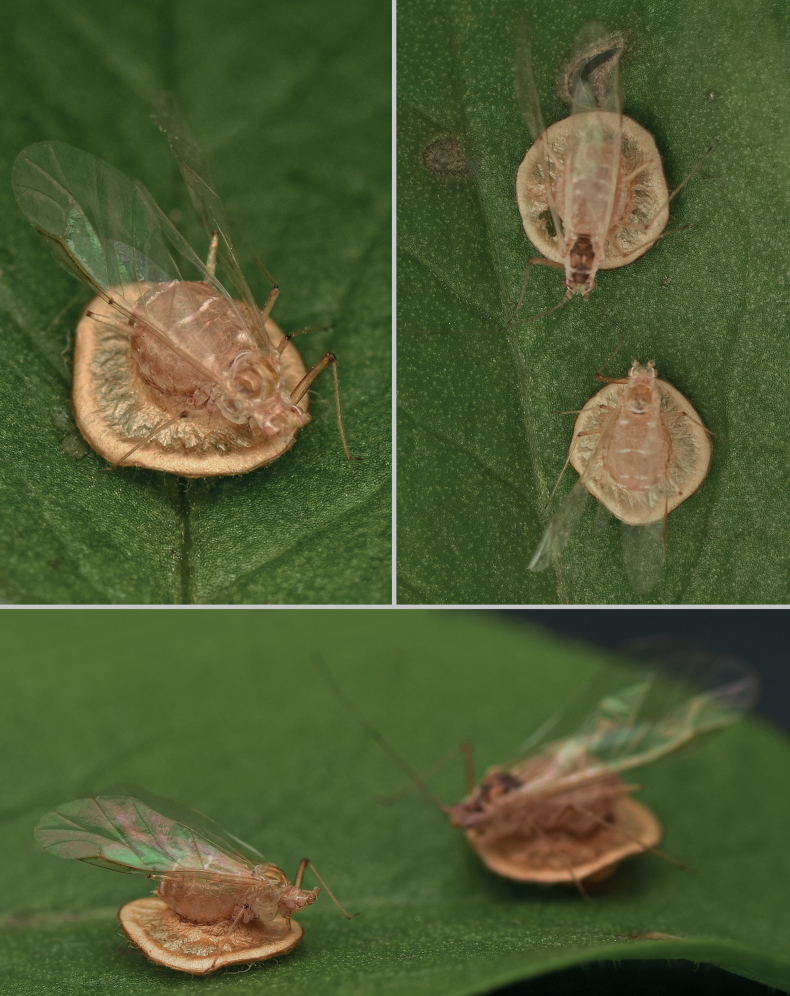
Aphid mummies of the new species, *Dyscrituluseuropaeus* sp. nov.

Figs 1A–H , 2A–H , 3

##### Material.

***Holotype***: 1♀, Spain, Málaga, Ronda, Sra Nieves, 1500 m alt., 4 June 1999, M. J. Gijswijt leg., found on *Acer* sp., collected by sweep net. ***Paratypes***: 1♀, same data as for holoype; 1♀, France, Mt. Ventoux, Plan de Perrache, 4 July 1995, M. J. Gijswijt leg., collected by sweep net; 3♀4♂, Serbia, Belgrade, New Belgrade, 5 June 2006, reared from mummies found on *A.pseudoplatanus*; 1♀, Serbia, Kruševac, Slobodište, 5 July 2013, reared from *D.platanoidis* on *A.pseudoplatanus*; 1♀4♂, Serbia, Belgrade, Pionirski Park, 28 May 2007, reared from mummies found on *A.pseudoplatanus*.Deposition: Holotype and two paratypes (from Spain and France) deposited in Naturalis Biodiversity Center, Leiden, Netherlands. Paratypes from Serbia deposited in collection of Institute of Zoology, University of Belgrade – Faculty of Biology, Belgrade, Serbia.

##### Diagnosis.

*Dyscrituluseuropaeus* sp. nov. can easily be distinguished from most commonly found species *D.planiceps* by having 21 antennomeres (Fig. 1B) (in one female specimen apical antennomera is half-divided, giving the impression of 22 antennomeres) (while *D.planiceps* has 23–24), coloration of first (F_1_) and second (F_2_) flagellomere (Fig. 1C) (F_1_ and first half of F_2_ yellow, while in *D.planiceps* F_1_, F_2_ and first half of F3 are yellow), shorter R1 vein (Fig. 1H) (pterostigma/R1 length ratio is 2.0–2.2 vs 1.5–1.6 in *D.planiceps*), more elongated petiole (Fig. 1F) (ratio of petiole length and width at spiracles level is 1.7 vs 1.5 in *D.planiceps*) and narrower ovipositor sheath (Fig. 1G). From *D.pygmaeus* and *D.dzhungaricus* it is differentiated by number of antennomeres (21 vs 15 in both *D.pygmaeus* and *D.dzhungaricus*) and presence of areola on propodeum. It is most similar to *D.trjapitzini*, however it differs in following morphological characters: number of antennomeres (21 vs 22 in *D.trjapitzini*), more elongated petiole (ratio of petiole length and width at spiracles level is 1.7 vs 1.5 in *D.trjapitzini*), color of F_1_ and F_2_ (F_1_ and half of F_2_ yellow vs F_1_ and F_2_ entirely yellow with darker apices in *D.trjapitzini*) and shorter F_1_ (4.5–4.6 length to width ratio vs 5 in *D.trjapitzini*).

##### Description.

**Female. *Head*.** (Fig. 1A) Head sparsely setose, wider than mesosoma at tegulae (head/mesoscutum width ratio 1.35–1.45). Eyes oval, relatively small. Clypeus sparsely setose. Tentorial index (tentoriocular line/intertentorial line) 0.25–0.32. Malar space equal to 0.18–0.22 of longitudinal eye diameter. Maxillary palps with 4 very long palpomeres, labial palps with 3 palpomeres. Antennae filiform, with 21 antennomeres (Fig. 1B), one female with half-divided apical antennomera. Flagellomeres cylindrical, with semi-erect setae subequal to flagellomere diameter. First (F_1_) and second (F_2_) flagellelomere 4.5–4.6 and 3.7–3.95 times as long as wide, respectively (Fig. 1C). F_1_ 1.15–1.28 times longer than F_2_. Both F_1_ and F_2_ without longitudinal placodes.

***Mesosoma*.** Mesoscutum with wide, deep notaulices, almost reaching prescutellar groove, dividing mesoscutum into three lobes (Fig. 1D). In lighter-colored specimens notaulices are paler and give an impression of reaching only half of mesoscutum. Two longitudinal rows of setae are present along the sides of notaulices. Lateral lobes of mesoscutum are covered with short dense setae at the proximity of prescutellar groove, which is deep and smooth. Scutellum with somewhat longer setae along the outer margins. Propodeum areolated, with distinct central areola, in some specimens with slightly irregular lateral carinae (Fig. 1E). In smaller specimens, central areola is somewhat wider. External areolae with 4–5 short setae on each side, dentiparal areolae smooth or with one long seta. Fore wing with marginal setae that are longer than the surface setae (Fig. 1H). Pterostigma triangular, 3.1–3.5 times as long as wide. Vein R1 (=metacarpus) shorter than pterostigma (pterostigma length/R1 vein ratio 2.0–2.2). Vein r&RS distinctly colored in the proximal part, subequal to R1 vein length (1.0–1.15); the rest of the r&RS is colorless, reaching almost to the outer margin of the wing. Veins m-cu and 2M are colored throughout.

***Metasoma*.** Petiole convex, 1.7 times as long as wide at spiracle level, with distinctly prominent transversal and longitudinal carinae (Fig. 1F). One central short longitudinal carina is feebly visible. On dorso-lateral sides two prominent longitudinal carinae are visible. Spiracular tubercles located closer to the anterior part of the petiole. Along the sides of the petiole, 4–5 long setae are present. Ovipositor sheaths elongated (2.2–2.4 length/width ratio), narrowed towards the apices, with 4–5 short setae across dorsal and ventral sides (Fig. 1G). Several campaniform sensillae situated at apical portion of ovipositor sheaths.

***Colour*.** Upper part of head is brown, lower part, clypeus and mouthparts yellow (except for darker apices of mandibles). Scape, pedicel and annellus are yellow. F_1_ is almost entirely yellow, except for narrow darker ring at apex; first half of F_2_ yellow, second half is brown. Remainder of antennae dark brown. Mesoscutum and propodeum brown, petiole light brown. Legs yellow. Metasoma (=abdomen) and ovipositor sheaths brown. Fore wing venation brown.

***Body length*.** 2.1 mm.

**Male.** Head with slightly larger eyes than in female (Fig. 2A). Tentorial index (tentoriocular line/intertentorial line) 0.35. Malar space equal to 0.21 of longitudinal eye diameter. Maxillary palps with 4 very long palpomeres, labial palps with 3 palpomeres. Antennae filiform with 23 antennomeres, stouter than in female (Fig. 2B). F_1_ and F_2_ are subequal, 1.8–1.9 as long as wide, bearing 8 and 9 longitudinal placodes, respectively (Fig. 2C). Mesoscutum with slightly shorter notaulices than in female (Fig. 2D). Propodeum with central areola (Fig. 2E). External areolae with 3–4 setae, dentiparal areolae smooth. Petiole 1.6 times as long as wide, with protrudent spiracles (Fig. 2F). Pterostigma 2.9–3.0 as long as wide; R1 vein longer than in female (pterostigma/R1 vein length ratio 1.78–1.79) (Fig. 2H). Genitalia as in Figure 2G. Body generally darker than in female, flagellomeres entirely brown.

***Body length*.** 1.9 mm.

##### Etymology.

The name of the new species is derived from its current distribution.

##### Distribution.

Europe.

##### Aphid host.

*Drepanosiphumplatanoidis* on *Acerpseudoplatanus* and *Acer* spp.

##### Note.

The morphology of the cocoon (Fig. 3) is typical for the genus, with external pupation, as described by Starý (1959).

### ﻿Key to the known species of *Dyscritulus* based on the females

**Table:** 

1	Number of antennomeres less than 20; propodeum without areola, with short diverging carinae at the posterior part (Fig. 4A)	**2**
–	Number of antennomeres more than 20 (Figs 1B, 5B); propodeum with complete central areola (Figs 1E, 5E)	**3**
2	R1 equal to half of pterostigma length; European distribution	***D.pygmaeus***
–	R1 extremely short, equal to 1/5 of pterostigma length (Fig. 4D); distributed in Kazakhstan, Russian Siberia and Mongolia	***D.dzhungaricus***
3	Petiole 1.7 times as long as wide at spiracle level (Fig. 1F); first half of F_2_ yellow and remainder dark brown; F_1_ 4.5–4.6 times as long as wide (Fig. 1C); number of antennomeres 21 (Fig. 1B); distributed in South Europe	***D.europaeus* sp. nov.**
–	Petiole 1.4–1.5 times as long as wide (Figs 4C, 5F); F_2_ entirely yellow; F_1_ at least 5.0 times as long as wide (Figs 4B, 5C); number of antennomeres 22–24	**4**
4	Number of antennomeres 23–24 (Fig. 5B); at least half of F_3_ yellow; European distribution	***D.planiceps***
–	Number of antennomeres 22, entire F_3_ dark brown; distributed in Western Caucasus	***D.trjapitzini***

Although male specimens of *D.pygmaeus* are unknown, other male species can easily be differentiated by the number of antennal segments: *D.dzhungaricus*, *D.europaeus* sp. nov., *D.trjapitzini* and *D.planiceps* have 17–19, 23, 24 and 25–26 antennal segments, respectively.

**Figure 4.**
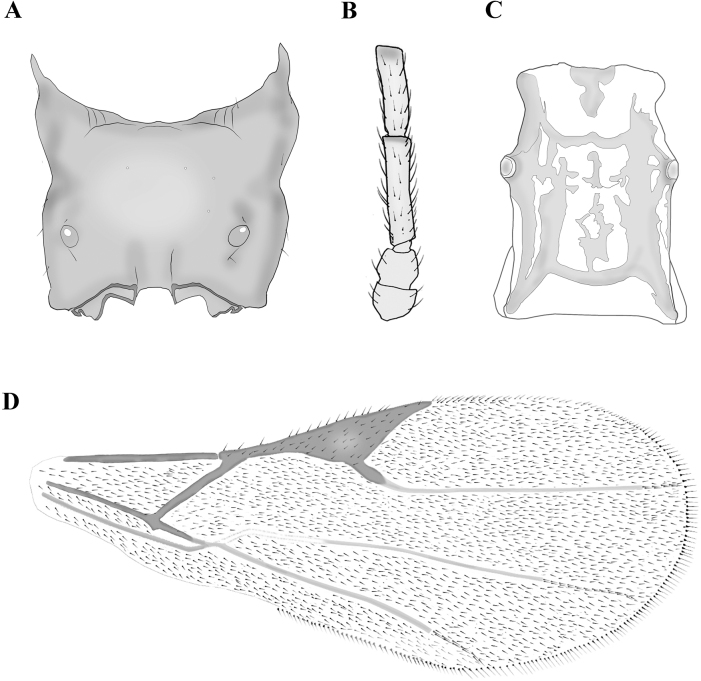
*Dyscritulusdzhungaricus* (**A, D**) and *D.trjapitzini* (**B, C**), not to scale **A** propodeum– dorsal aspect **B** scape, pedicel, and F_1_ and F_2_ flagellomeres **C** petiole – dorsal aspect **D** fore wing. Redrawn from Davidian 2018, 2019.

**Figure 5.**
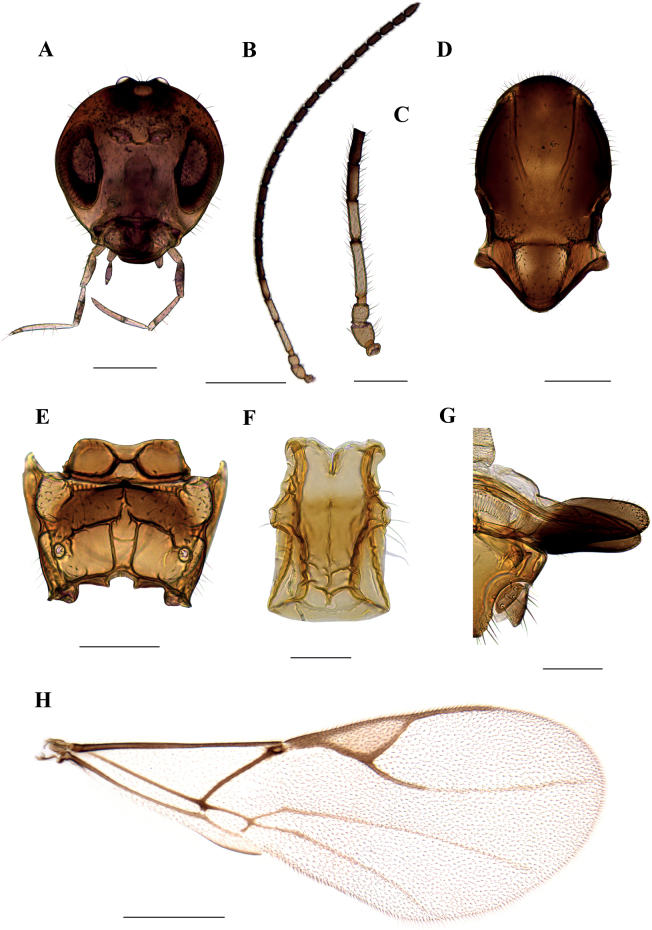
*Dyscritulusplaniceps* female **A** head **B** antenna **C** scape, pedicel, and F_1_–F_4_ flagellomeres **D** mesonotum (=mesoscutum) – dorsal aspect **E** propodeum – dorsal aspect **F** petiole – dorsal aspect **G** ovipositor sheaths **H** fore wing. Scale bars: 200 µm (**A**, **C**–**E**); 500 µm (**B**, **H**); 100 µm (**F**, **G**).

## ﻿Discussion

Throughout the last two centuries, museum and other institutional collections were considered important components of research, particularly in the field of taxonomy and systematics (Suarez and Tsutsui 2004). During the past two decades, museum collections have played an important role in identifying ecological responses to global change and habitat loss (Pyke and Ehrlich 2010; Kharouba et al. 2019). For the study of the subfamily Aphidiinae, the specimens preserved in museum collections are of crucial importance. Rare Aphidiinae species, holotype and paratype specimens used for research of taxonomy, systematics, morphology and phylogeny of the group are frequently acquired from museums and institutional collections (Petrović 2022). Recently, an increasing number of species have been described based on specimen loans (Davidian 2016; Tomanović et al. 2020; Petrović et al. 2021). Moreover, a member of *Dyscritulus* (*D.dzhungaricus*) was described from the collection of the Zoological Institute of the Russian Academy of Sciences (Davidian 2018). The first specimens of *D.europaeus* sp. nov. were also discovered in the museum collections of the Naturalis Biodiversity Center: only after re-examination of material previously identified as *D.planiceps* were additional individuals belonging to this species found.

The phylogenetic position of *Dyscritulus* within the tribe Praini is uncertain. The results of parsimony analysis, which considered both morphological and life history characters of members of Praini, grouped *Dyscritulus* together with *Areopraon* and *Pseudopraon* as monophyletic, and positioned *Dyscritulus* as a sister group to these two genera (Tomanović et al. 2006). Several studies have included *D.planiceps* in molecular phylogenetic analysis at the family (Dowton et al. 2002) or subfamily (Kambhampati et al. 2000; Sanchis et al. 2000) level. However, the tribe Praini was, besides *Dyscritulus*, represented only by genus *Praon* (Sanchis et al. 2000; Dowton et al. 2002) or genera *Praon* and *Pseudopraon* (Kambhampati et al. 2000).

Except for *D.planiceps* and *D.europaeus* sp. nov., the three remaining species of *Dyscritulus* have unknown aphid hosts. *Dyscritulustrjapitzini* was reared from mummies found on *Quercus* L. and *Fagusorientalis* Lipsky. It might be that *D.trjapitzini* parasitizes some other aphid genera, or that the aphid host belonging to *Drepanosiphum* or *Periphyllus* was mummified on *Quercus* sp. and *F.orientalis* by accident. *Dyscrituluseuropaeus* sp. nov. originates from *Drepanosiphum* aphids (*D.platanoidis*) or aphid mummies collected on *Acer*.

While *D.planiceps* is distributed throughout Europe, *D.pygmaeus* has been recorded only twice since its description, in Finland and Hungary (Hellen 1971; Polgar 1983). *Dyscritulustrjapitzini* is known only from its type locality Georgia (Abkhazia) (Davidian 2018) and *D.dzhungaricus* is described from Kazakhstan (Davidian 2019) and also reported from Russian Siberia and Mongolia (Davidian 2020), In this article, the new species has a southern European distribution, having been recorded in Spain (Málaga), Serbia and France (Mt. Ventoux, southern France). However, the possibility that its distribution range spans across entire Europe is not excluded. According to the currently available data, *D.planiceps* and *D.europaeus* sp. nov. are sympatric in southern Europe. Nevertheless, the revision of the available material should clarify their true distribution. As in our case, where important morphological differences were overlooked and samples were initially identified as *D.planiceps*, it is possible that material preserved in other collections also contains both species.

Although heavy infestations of *Drepanosiphum* aphids do not kill sycamore and maple trees, they significantly affect their health and appearance. With heavy infestations, trees produce smaller leaves at maturity and the growth of the stem wood is reduced (Dixon 1971). It has been shown that in the absence of aphids, sycamore could achieve nearly twice their normal annual growth. In addition, severely attacked leaves succumb to chlorosis, and the heavily produced honeydew covers the leaves and provides a suitable surface for the development of sooty moulds (Speight 1980). In urban areas, chemical control using insecticides is generally avoided. Among the natural enemies, parasitoids play an important role in regulating size of an aphid population. *Aphidiussetiger* Mackauer, 1961, *Trioxyscirsii* Curtis, 1831, *T.falcatus* Mackauer, 1959, *T.acericola* Starý & Mackauer, 1971 and *Falciconuspseudoplatani* Marshall, 1896 are commonly found parasitizing aphids on *Acer* trees (Kavallieratos et al. 2016). Compared to them *Dyscritulus* specimens are collected rather rarely. The probable reason is their highly specialized life cycle and specific seasonal activity. The new species is an additional member of a highly specialized group of *Drepanosiphum* parasitoids that may contribute to the natural control of aphid outbreaks. The holotype and paratype specimens of *D.europaeus* sp. nov. were collected in both urban and protected areas (national parks), i.e., on cultivated and forest trees. Even after its revision (Starý 1959) and additional species descriptions (Davidian 2018, 2019), the biology of the genus still remains poorly known. It is likely that these five currently known species represent only a fraction of its actual diversity. Accordingly, further efforts should be made towards revealing the distribution, trophic associations and life cycle of the genus. Likewise, an integrative approach of morphological and molecular methods with a sufficient sample size should reveal the phylogenetic relationships among congeners.

## Supplementary Material

XML Treatment for Dyscritulus europaeus

## ﻿Additional information

### Author ORCIDs

Korana Kocić https://orcid.org/0000-0002-0926-1595

Andjeljko Petrović https://orcid.org/0000-0002-8126-9620

Jelisaveta Čkrkić https://orcid.org/0000-0002-4547-1346

Cornelis van Achterberg https://orcid.org/0000-0002-6495-4853

Željko Tomanović https://orcid.org/0000-0002-5063-5480

### Data availability

All of the data that support the findings of this study are available in the main text.
